# Enrichment of Minor Alleles of Common SNPs and Improved Risk Prediction for Parkinson's Disease

**DOI:** 10.1371/journal.pone.0133421

**Published:** 2015-07-24

**Authors:** Zuobin Zhu, Dejian Yuan, Denghui Luo, Xitong Lu, Shi Huang

**Affiliations:** State Key Laboratory of Medical Genetics, Central South University, 110 Xiangya Road, Changsha, Hunan, 410078, China; Duke University, UNITED STATES

## Abstract

Parkinson disease (PD) is the second most common neurodegenerative disorder in the aged population and thought to involve many genetic loci. While a number of individual single nucleotide polymorphisms (SNPs) have been linked with PD, many remain to be found and no known markers or combinations of them have a useful predictive value for sporadic PD cases. The collective effects of genome wide minor alleles of common SNPs, or the minor allele content (MAC) in an individual, have recently been shown to be linked with quantitative variations of numerous complex traits in model organisms with higher MAC more likely linked with lower fitness. Here we found that PD cases had higher MAC than matched controls. A set of 37564 SNPs with MA (MAF < 0.4) more common in cases (P < 0.05) was found to have the best predictive accuracy. A weighted risk score calculated by using this set can predict 2% of PD cases (100% specificity), which is comparable to using familial PD genes to identify familial PD cases. These results suggest a novel genetic component in PD and provide a useful genetic method to identify a small fraction of PD cases.

## Introduction

Parkinson disease (PD) is the second most common neurodegenerative disorder in the aged population, with a prevalence of 1–3% over 70 years of age[1]. However, unlike diabetes or cardiovascular disease, there are no blood markers that can be used to predict risk for PD. It is now well established that Parkinson’s disease (PD) contains a genetic component. Genes for familial forms of PD have been discovered, including *LRRK2* [2–6], *SNCA*[7,8], *PINK1*[9], *PARK*2[10], *DJ-1*[11,12], *VPS35*[13,14] and *ATP13A2*[15,16]. However, most PD cases are sporadic and no known mutations have been found to account for even a small fraction of the disease.

Genome-wide association studies (GWAS) have found some success in identifying ~26 PD susceptibility loci, but their roles in the disease largely remain unclear[17]. There has been growing debate over the nature of the genetic contribution to individual susceptibility to common complex diseases[18–20]. The most difficult problem is that most of the associated SNPs captured by GWAS have very small effect sizes and the proportion of heritability explained is at best modest for most traits/diseases[21–23]. Many of the rare coding variants that are associated with diseases and predicted to be damaging also appear in healthy controls[23].

Recent studies have begun to show that a much larger than expected portion of the human genome may be functional [24–29]. Most of the risk alleles identified from hundreds of GWAS of human diseases are minor allele(MAs)[30]. We have recently shown that the collective effects of whole genome wide collection of MAs are linked with lower reproductive fitness in *C*.*elegans* and yeasts[28]. Such findings are to be intuitively expected as the origins of MAs are random errors or mutations. An organism can certainly accommodate some limited amounts of random variations within its building parts or DNAs, but too much random errors or mutations may exceed an organism’s maximum level of tolerable disorder or entropy. Thus overall level of randomness or MA amounts may be expected to be higher in complex diseases relative to controls.

Researchers have used a set of susceptibility loci to create a genetic risk score to better predict PD risk[31,32]. But these predictions were generally poor and not meaningful for clinical use, likely because the susceptibility loci used are only a small part of the total genetic contribution to PD. These prediction models have calculated the area under the receptor-operator curve (AUC). But they generally did not consider or could not generate meaningful true positive rate (TPR) with 100% specificity (no false positives), a more useful measure in clinical applications.

Here we studied the overall level of randomness in PD cases relative to controls as measured by total MA amounts in an individual. We show that PD cases had higher total amount of MAs. We further identified a set of MAs that can produce good AUC and TPR scores both in an internal 10-fold cross-validation experiment and an external cross-validation experiment. Our prediction model can predict ~2% PD cases with 100% specificity by using only genetic information.

## Materials and Methods

### Cohort description

Two GWAS case control datasets of PD, NeuroGenetics Research Consortium cohort(phs000196.v2.p1) [33] and Autopsy-Confirmed Parkinson Disease GWAS Consortium cohort(phs000394.v1.p1), were downloaded from database of Genotypes and Phenotypes (dbGaP). All the case-control subjects were whites. All cases were evaluated by a neurologist and each participant underwent a detailed evaluation for Parkinson's disease, and met either the Gelb criteria or the UK Brain Bank Criteria. All controls must be of no clinical diagnosis of parkinsonism and no neurologic disorder at enrollment, by self-report or exam. These subjects were scanned for ~900K common SNPs using Illumina HumanOmni1-Quad_v1-0_B. Principal component analysis (PCA) using the GCTA tool was used to estimate the genetic relatedness [34]. Outliers were excluded by principle component 1–3. Duplicated individuals were excluded from the analysis. All analyses were done with autosomal SNPs. Genotype distributions for each SNP were consistent with Hardy-Weinberg equilibrium (P > 0.01). After outlier exclusion, phs000196.v2.p1 cohort has 1999 cases and 1986 controls and the phs000394.v1.p1 cohort has 609 cases and 305 controls (S1 Table). Summary statistics describing the two datasets are provided in Table 1.

**Table 1:** Description of cohorts.

Description	phs000196.v2.p1		phs000394.v1.p1		phs000674.v1.p1
	Case	Control	Case	Control	control
Number of participants	1999	1986	609	306	37441
Number of SNPs	857662	857662	857662	857662	670176
Age at collection	67.26(10.67)	70.32(14.09)	-	-	-
Male(%)	67.3	33.7	72.8	27.2	42.9
Age of onset	58.34(12.09)	-	-	-	-
Age at death	77.3(7.43)	87.78(7.85)	77.52(8.47)	81.76(12.72)	-

Standard deviations are given in parentheses. The molecular data of the two PD cohorts(phs000196.v2.p1 and phs000394.v1.p1) were both genotyped from the platform of Illumina-HumanOmni1-Quad_v1-0_B. The phs000674.v1.p1 cohort was genotyped from the platform Affymetrix-Axiom_KP_UCSF_EUR.

In addition, to test the predictive power of the PD specific set of 37564 SNPs identified here, we used a control population containing 37441 white Europeans or European Americans (phs000674.v1.p1, Resource for Genetic Epidemiology Research on Adult Health and Aging)[35]. Summary statistics describing this dataset are also provided in Table 1.

### Statistical analysis

Minor allele status of each SNP was determined by calculating MAF using the control dataset. The MAF of each SNP was calculated by PLINK and SNP Tools for Microsoft Excel[36,37]. From MAF data of 1986 controls, we obtained the MA set, which excluded non-informative SNPs with MAF = 0 in both cases and controls or with MAF = 0.5 in controls. The MA set was equivalent to an imagined individual who is homozygous for all the MAs of informative SNPs analyzed. Risk profiles were generated from 1999 cases and 1986 controls. All the SNPs with >5% missing data or MAF <10–4 were excluded. The p-values of each SNP were calculated by PLINK. The same MA set was used for analyzing the other case control dataset in the external cross validation test.

Minor allele content (MAC) means the ratio of the number of minor alleles divided by the total number of SNPs scanned (non-informative NN SNPs were excluded). The MAC of each individual was scored using a custom script (S2 Script). Each MA was given a weighted risk score calculated by logistic regression test, which was equal to the coefficient of the logistic regression test. For heterozygous MAs, the weighted risk score was 0.5 x the coefficient. The total MA numbers of each individual were then converted to a total weighted risk score by summing up the coefficient of each MA by using a custom script (S2 Script).

### Haplotype construction

Haplotype block estimation were phased using PLINK with pairwise LD calculated for SNPs within 200kb. Haplotype selection was performed as described previously[38]. A standard logistic regression was performed on all SNPs of each haplotype to obtain association significance with the disease. For each haplotype, a representative SNP with the best disease linkage was chosen for risk prediction analysis and all SNPs chosen must satisfy the minimal selection criterion of MAF<0.4 and *P* < 0.05.

### Risk prediction

We performed two types of cross-validation experiments. For an external cross-validation analysis, the phs000196.v2.p1 cohort was used as a training set, and testing was performed on the phs000394.v1.p1 dataset. Each experiment’s discriminatory capability was evaluated using the receiver operating characteristic (ROC) curve. We then calculated the area under the curve (AUC) and the true positive rate (TPR) using Prism5. TPR is the proportion of cases who had a risk score higher than that of any control individual. The AUC quantifies the overall ability of the test to discriminate between cases and controls. A truly useless test (one no better at identifying true positives than flipping a coin) has an area of 0.5. A perfect test (one that has zero false positives and zero false negatives) has an area of 1.00.

In order to obtain a best MA set for risk prediction, six models were constructed using logistic regression. Five of these models were based on MAF, and the remaining one used haplotypes. We then obtained AUC of each set in the testing dataset phs000394.v1.p1.

In the internal 10 fold cross-validation analysis, the phs000196.v2.p1 cohort was randomly partitioned into 10 subsamples. Of the 10 subsamples, a single subsample is retained as the validation data for testing the model, and the remaining 9 subsamples are used as training data. The cross-validation process was then repeated 10 times, with each of the K subsamples used exactly once as the validation data. The 10 results were averaged to produce a single estimation. 10-fold cross-validation is commonly used and the advantage of this method over repeated random sub-sampling is that all observations are used for both training and validation, and each observation is used for validation exactly once.

Since the external cross validation analysis above identified the best MA set as having MAF < 0.4, we only analyzed this set of MAs in this internal cross validation analysis. This set has 37564 SNPs.

## Results

### Enrichment of minor alleles in PD

**Fig 1:**
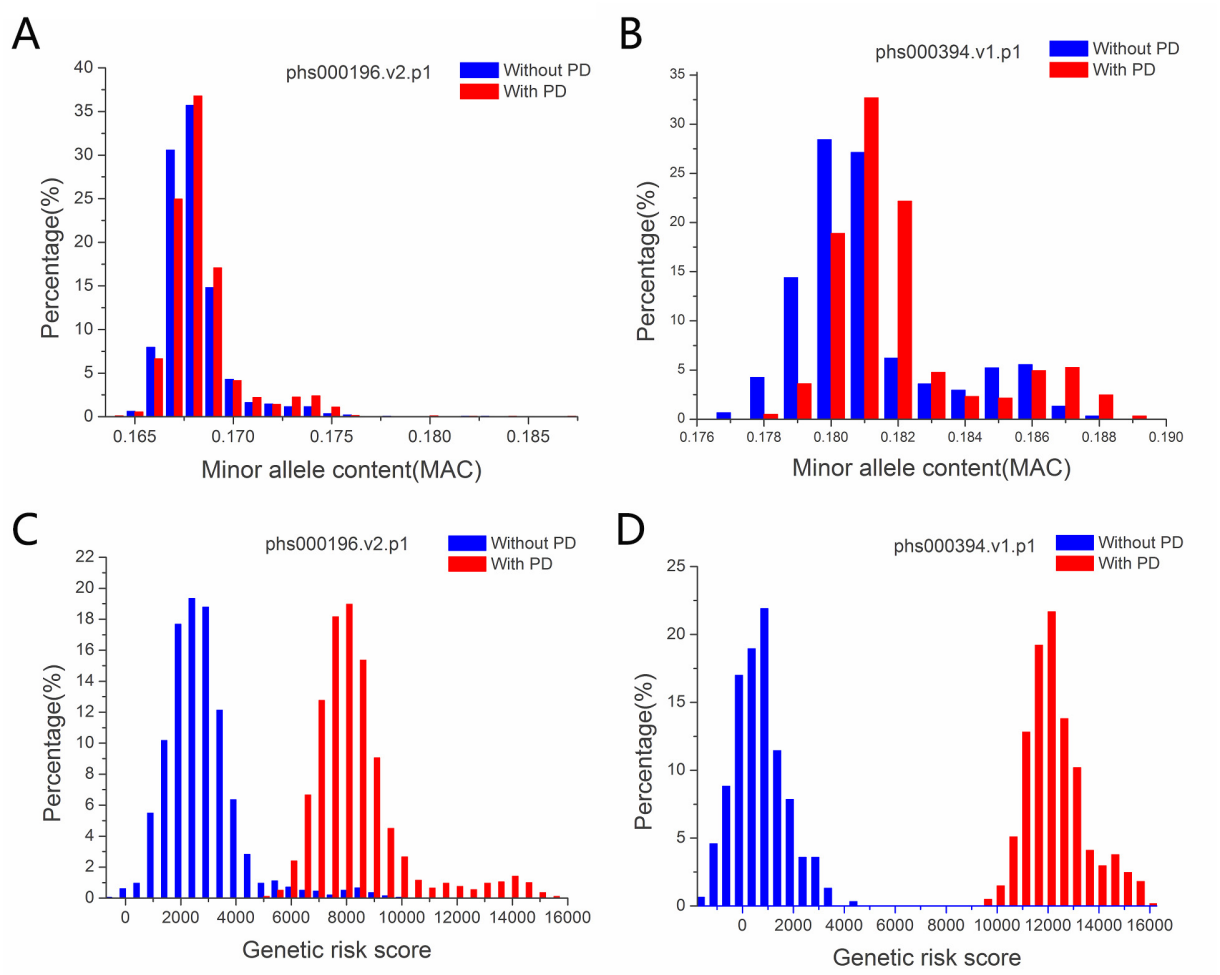
Distribution of MAC and genetic risk allele scores of MAs by case–control status. MAC: Minor allele content of SNPs with MAF < 0.4. Genetic risk score, the total risk score of all the MAs in an individual by adding the coefficient of logistic regression test of each MA.

SNPs typically have just two alleles in a population and the minor allele (MA) has frequency (MAF) <0.5. Here we calculated the minor allele content of each individual with MA defined as those with MAF < 0.4 in a control population. SNPs with MAF >0.4 and <0.5 were not considered in order to be more certain about the MA status. Using the control population of the phs000196.v2.p1 dataset, we obtained the MA status of each SNP and calculated the MAC of each individual control and case. We found that PD cases had higher average MAC than the controls (p = 4E-09, one-way ANOVA) (S2 Table). To confirm this finding, we examined a second case control dataset phs000394.v1.p1. From the control population in the second dataset, we obtained the MA status of each SNP and calculated the MAC of each individual control and case. Again, cases were found to have higher average MAC than the controls (p = 1E-08, one-way ANOVA) (S2 Table). For both datasets, the MAC was both normally distributed with the distribution of cases slightly shifted to the right or higher MAC position (Fig 1A and 1B).

Using logistic regression analysis, we obtained a coefficient score for each SNP, which was termed the weighted risk score of each SNP. The total MA number of each individual was then converted into a total weighted risk score by adding the coefficient of each MA (major alleles were not counted). By converting MAC into the weighted risk scores could almost entirely separate the cases from the controls for both datasets (Fig 1C and 1D).

**Fig 2:**
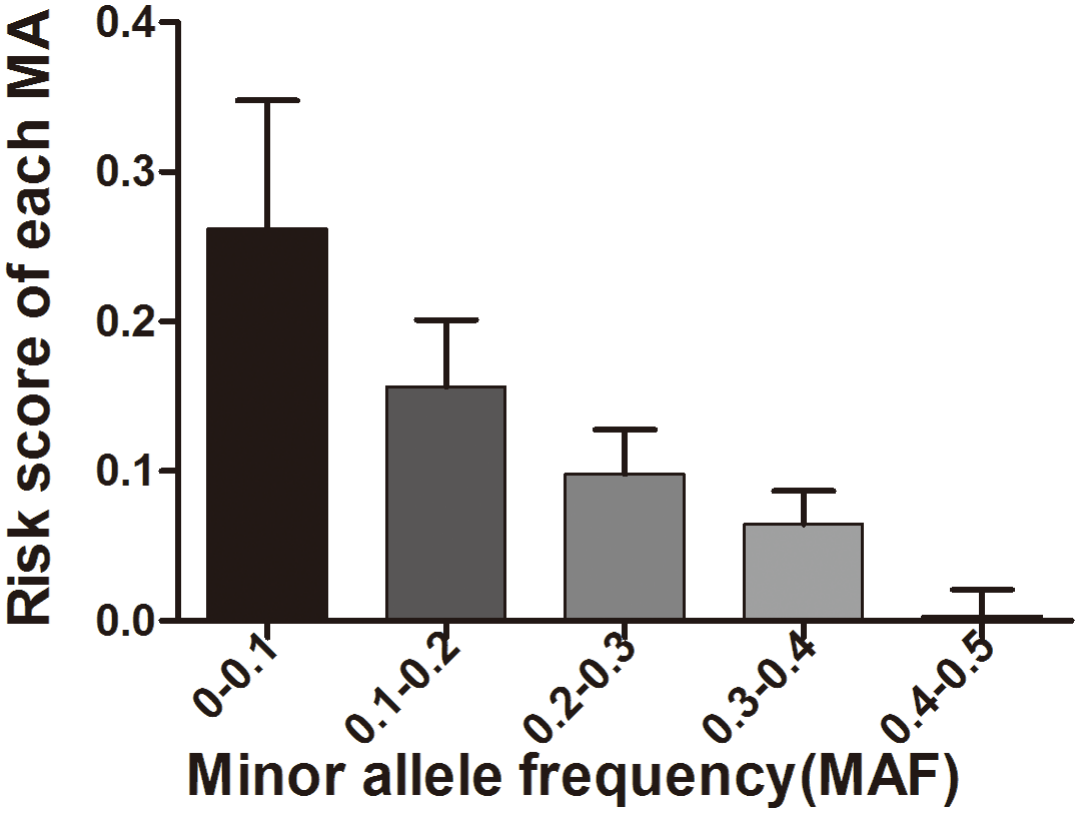
Correlation between MAF and PD risk score. Shown are the average risk score for each category of MAs as classified by MAF.

To further characterize the role of MAs in PD. We divided SNPs into five groups by their MAF. For each group of SNPs, we calculated the total weighted risk score of each individual, and obtained the average risk score of each SNP by dividing the total score by the number of SNPs. The result showed progressively higher risk scores for lower MAF SNPs (Fig 2), indicating that low frequency SNPs may be under more negative natural selection as a result of carrying higher risk of diseases.

### Risk prediction

**Fig 3:**
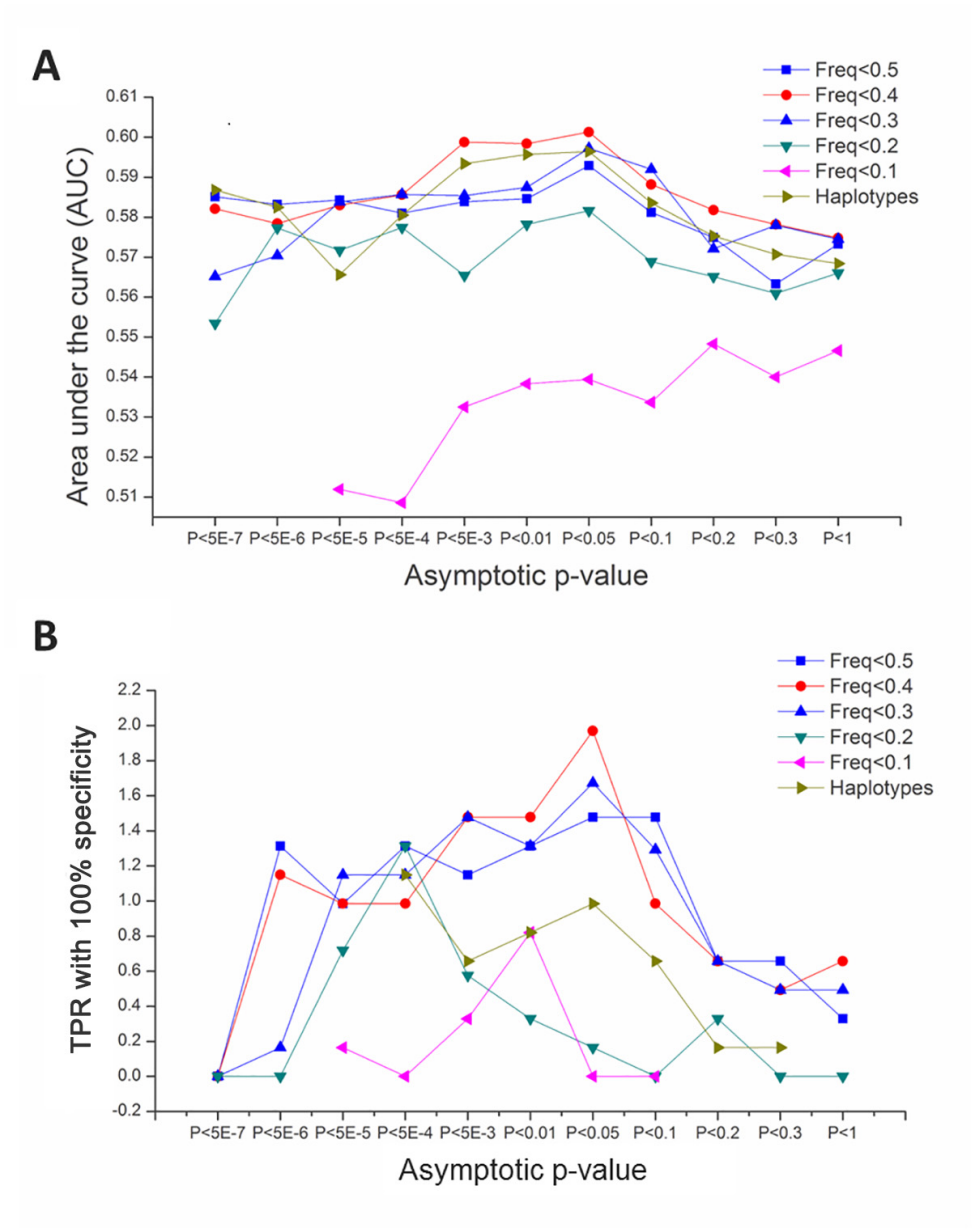
Discriminatory ability of different prediction models. SNPs were divided into 6 models based on MAF or haplotype. AUC (A) and TPR (B) were calculated using a training dataset and a test dataset. Each model was examined using MAs with different asymptotic P-value from the logistic regression test.

Our next goal was to obtain a PD specific set of MAs from a training dataset and use it to perform an external cross-validation analysis, i.e., to predict PD status for an unrelated dataset (testing dataset). We chose the phs000196.v2.p1 dataset as the training dataset. In order to obtain a best MA set for risk prediction, six models were constructed using logistic regression. Five of these models were based on MAF, and the remaining one used haplotypes. We then used the receiver operater characteristic (ROC) curve to examine the discriminatory capability or AUC of each set in the testing dataset phs000394.v1.p1. The set showing the largest AUC as well as TPR was the one with MAF < 0.4 with each MAs’ linkage significance passing the threshold of P<0.05 (Fig 3, S3 Table). The AUC for this set is 0.60 (95%CI: 0.5612–0.6414) and the TPR is 2.0%, (95%CI:1.022%-3.417%).

We further performed a 10 fold internal cross-validation analysis using the above training phs000196.v2.p1 dataset. Since the external cross validation analysis above identified the best set as having MAF < 0.4 with each MA passing the threshold of P < 0.05, we only analyzed this set of MAs in this internal cross validation analysis, which has 37564 MAs (see S4 Table for details of this set in terms of risk scores of each individual SNP in the set). For this set of 37564 SNPs, we obtained an average AUC of 0.60 (95%CI, 0.5573–0.6425) and TPR of 2.0% (95%CI,1.233%-2.699%), which was similar to the above results of external cross validation analysis.

This set of 37564 SNPs was then tested in a large control population (phs000674.v1.p1), containing 37441 white Europeans or European Americans[35]. Of the 37564 MAs or SNPs, only 8994 were shared between this control dataset and the PD dataset phs000196.v2.p1. Using these shared MAs, we calculated the MA risk scores of each control individual as well as each PD individual. There were 1.2% PD cases with a risk score greater than 745, whereas all controls (1986 controls from phs000196.v2.p1 and the 37441 controls from phs000674.v1.p1) had scores less than 745 (S5 Table). Therefore, even with less power due to loss of ~75% of MAs in the original set, the PD specific set of MAs produced only a slightly poorer TPR when using a different control dataset.

## Discussion

The pathogenesis of PD is multifactorial and includes a strong genetic component. It is well established that complex diseases cannot be explained by a small number of rare variants with large effects[21]. But research on common small effects variants has had little progress. This is likely because of a long-standing lack of appreciation for the deleterious effects of too many random errors at the genomic level. Here we show indeed that having too many minor alleles at the genomic level may be a novel genetic factor for PD. While the control datasets had less number of males, we did not find any significant sex bias in the distribution of minor alleles. Our results here add to the long list of traits linked with MAC[28,39,40]. By identifying a PD specific set of 37564 MAs, the study here suggests that different traits or diseases may be linked with different sets of MAs, although it cannot exclude the likely possibility that different disease-specific sets may share a fraction of SNPs. Thus, while the number of SNPs involved in a disease may be quite large as indicated by this work here, much larger than expected from previous studies, it may not mean a lack of disease specificity in the collective effects of SNPs.

Our finding of higher MAC in PD cases is not expected by known works on PD. Published PD risk SNPs are relatively few in numbers. Therefore even if these known risk alleles are mostly minor alleles, it may not predict that cases should have more MAs when a genome wide collection of ~1 million SNPs are considered. If most MAs are not related to PD except those few published PD alleles, then the average MAC of cases should not be significantly different from the controls. In fact, while most bench biologists have thought otherwise, nearly all in the population genetics field still believe that most SNPs are neutral or that most MAs are minor because of random drift rather than because of disease-association.

The findings of higher MAC in PD cases is consistent with our intuitive hypothesis that a highly complex and ordered system such as the human brain must have an optimum limit on the level of randomness or entropy in its building parts or DNAs. Too much randomness over a critical threshold may trigger complex diseases. There may be only one unique and optimum way to build a complex system but there could be numerous ways to break it. While it may only take one single major effect error in a major pathway to cause diseases, it would require the collective effects of a large number of minor effect errors in many different pathways to achieve a similar outcome.

Negative selection by way of common diseases such as PD may be one of the ways to maintain a maximum or optimum limit on genomic entropy and to render the disease risk alleles minor ones in the population. Although complex diseases tend to be late onset and hence well past the age of reproduction, which may be expected to have little selective effects on genes, one can still envision several ways for late onset common diseases to prevent accumulation of disease risk alleles in a population. First, an elderly patient is a huge burden to a family and may negatively impact the young family members both economically and emotionally in their competitive advantage to succeed. Second, an elderly patient may not provide much help to young family members, which would make them less competitive relative to young people with healthy parents or grandparents. Third, individuals with too many MAs may be already at a fitness disadvantage in many different traits including reproduction prior to disease onset at older age [28, 38, 39]. Finally, negative selection *en utero* may also explain the lower frequency of some of the risk MAs, and the deleterious effects of these MAs on some late onset diseases may reflect pleiotropy. Most known late onset disease genes are well known to play a role in early development. Thus, the minor nature of some of these MAs linked with late onset diseases such as PD may reflect in part negative selection *en utero* rather than by these diseases *per se*.

It has become a standard practice to calculate AUC as a measurement of the prediction quality[41,42]. However, some authors have contended that the AUC is often of little practical use and may be insensitive to changes that would otherwise be considered important in a diagnostic setting[43–45]. Here, we have calculated AUC as an index to evaluate the prediction, which is comparable to other previous results. We also obtained a meaningful TPR of 2% that appears to be better than most previous studies on PD.

It has been reported that the prediction quality can be improved when there are a large number of SNPs, each of which is merely nominally significant, especially for diseases such as bipolar disorder and coronary heart disease[46]. Here, our prediction model for PD also follows this trend with the prediction quality peaking at the case-association significance threshold of P<0.05. It has been reported that a haplotype predictor is better than a predictor using all SNPs for Crohn’s disease[38]. But our haplotype predictor did not perform the best. This may be due to the fact of smaller number haplotypes relative to our best model that used 37564 SNPs. We used 6000 haplotypes with a LD block size of 200Kb. Although the use of haplotypes may prevent interferences by co-segregating SNPs, it may also remove PD linked SNPs as one haplotype may be further divided into more haplotypes and may contain several SNPs each independently linked with PD. Models using small number of SNPs may be more susceptible to influence by random effects, while using too large number of SNPs may contain many irrelevant SNPs. Thus a good predicative model may require a fine balance between high amounts of PD linked SNPs and low amounts of irrelevant SNPs.

Our best predictor model has a TPR value of 2% or could detect about 2% PD patients as verified by both external and internal cross-validation experiment. A slightly lower TPR of 1.2% was obtained when using a different control dataset and less number of informative SNPs, indicating that our method is robust. The value is similar to that using mutations in the most commonly mutated familial gene like *LRRK2* to detect familial cases in the PD population[2–6]. Therefore, while the absolute number of cases to be detected is still low relative to the total number of cases, it may still be significant to be potentially useful. It should be possible to further improve the method in future studies using larger sample sizes and larger number SNPs. Our method may also be similarly applied in other common diseases.

## Supporting Information

S1 Script The script for calculating MAC of each individual. (PL)

S2 Script The script for calculating the risk score of each individual. (PL)

S1 Table Principal component analysis (PCA). (XLSX)

S2 Table The MAC and risk scores of the individuals in the two case control datasets. (XLSX)

S3 Table Summary of AUC and TPR(100% specificity). (XLSX)

S4 Table MAs set for prediction. (XLSX)

S5 Table Risk scores of each individual using the large control population for validation test. (XLSX)
